# Middle-Aged *Lpaatδ*-Deficient Mice Have Altered Metabolic Measures

**DOI:** 10.3390/life12111717

**Published:** 2022-10-27

**Authors:** Michelle Victoria Tomczewski, Maria Fernanda Fernandes, Rajan Singh Grewal, Robin Elaine Duncan

**Affiliations:** Department of Kinesiology and Health Sciences, Faculty of Health, University of Waterloo, 200 University Ave W., BMH1044, Waterloo, ON N2L 3G1, Canada

**Keywords:** AGPAT4, LPAATδ, phosphatidic acid, indirect calorimetry, energy metabolism

## Abstract

Lysophosphatidic acid acyltransferases/acylglycerophosphate acyltransferases (LPAATs/AGPATs) are a group of homologous enzymes that catalyze the formation of phosphatidic acid (PA) from lysophosphatidic acid. We have previously reported that LPAATδ/AGPAT4 localizes to mitochondria, suggesting a potential role in energy metabolism. However, in prior studies of young *Lpaatδ*-deficient mice (age 9–12 weeks old), we found no differences in body weights, food intakes, activity levels, respiratory gas exchange, or energy expenditure compared to their wildtype (*Wt*) littermates. To test whether *Lpaatδ*^−/−^ mice may develop differences in metabolic measures with advancing age, we recorded body weights and food intakes, and used metabolic chambers to assess ambulatory and locomotor activity levels, oxygen consumption (VO_2_), carbon dioxide production (VCO_2_), respiratory exchange ratio (RER), and total energy expenditure (heat). Fourteen-month-old *Lpaatδ*^−/−^ mice had significantly lower mean body weights compared to *Wt* littermate controls (44.6 ± 1.08 g vs. 53.5 ± 0.42 g, respectively), but no significant differences in food intake or activity levels. This phenotypic difference was accompanied by significantly elevated 24 h daily, and 12 h light and dark photoperiod average VO_2_ (~20% higher) and VCO_2_ (~30% higher) measures, as well as higher RER and total energy expenditure (heat) values compared to *Wt* control littermates. Thus, an age-related metabolic phenotype is evident in *Lpaatδ*^−/−^ mice. Future studies should examine the role of the lipid-modifying enzyme LPAATδ across the lifespan for greater insight into its role in normal and pathophysiology.

## 1. Introduction

Lysophosphatidic acid acyltransferases/acylglycerophosphate acyltransferases (LPAAT/AGPAT) are a family of enzymes that function in the second step of the Kennedy pathway of glycerophospholipid and triacylglycerol (TAG) synthesis [1,2]. The Kennedy pathway begins with the acylation of glycerol-3-phosphate (G3P) by glycerol-3-phosphate acyltransferase (GPAT) enzymes to form lysophosphatidic acid (LPA), followed by LPAAT activity to convert LPA to phosphatidic acid (PA). The acyltransferase enzymes of the Kennedy pathway share some homology, but are encoded by different genes, and differ in substrate preferences, tissue expression, and subcellular localization, resulting in different roles for each in cells and in the body [3].

In total, five enzymes (LPAATα-ε/AGPAT1-5) have been identified as canonical LPAAT/AGPAT enzymes [3]. While all members of this family function primarily in the synthesis of PA from LPA, these enzymes are encoded by different genes, can localize to different subcellular regions, and provide metabolites that support apparently disparate down-stream lipid synthesis pathways, and are therefore not redundant [3]. Knockout mouse models have been generated for three of these enzymes, *Lpaatα*/*Agpat1* [4], *Lpaatβ*/*Agpat2* [5,6], and *Lpaatδ*/*Agpat4* [7,8,9]. In studies of mice deficient in *Lpaatα*/*Agpat1,* metabolic abnormalities were evident at a young age, including lower body weights and reduced adiposity, as well as more severe pathological changes including reduced post-weaning survival and the development of tonic-clonic seizures that could be fatal [4]. Mice deficient in *Lpaatβ*/*Agpat2* also manifest severe differences in early mortality and metabolic parameters. Like *Lpaatα*/*Agpat1* knockout mice, *Lpaatβ*/*Agpat2* knockout mice have high rates of neonatal mortality, although death tends to occur prior to weaning [5,6]. *Lpaatβ*/*Agpat2* knockout mice also exhibit a more severe metabolic phenotype than *Lpaatα*/*Agpat1* knockout mice, with the development of a generalized lipodystrophy.

Similar to other LPAAT/AGPAT enzymes, LPAATδ/AGPAT4 catalyzes the formation of PA using LPA as acyl acceptors and fatty acyl-CoAs as acyl donors [10]. However, unlike LPAATα/AGPAT1 or LPAATβ/AGPAT2, which are detected primarily in microsomes, this enzyme has been reported by our group to localize to the outer mitochondrial membrane [10], and by others to the endoplasmic reticulum (ER) [11], and the Golgi complex [12]. Our laboratory has studied the molecular and physiological role of LPAATδ using gene deficient mice (*Lpaatδ*^−/−^), and has found several differences from *Lpaatα*/*Agpat1* and *Lpaatβ*/*Agpat2* knockout mice, including an absence of neonatal lethality, but also much less pronounced metabolic differences at an early age [7,8,9,10,13]. 

In studies of young male and female *Lpaatδ*^−/−^ mice (ages 9–12 weeks) [9], we had expected to see decreases in body weights and adiposity, similar to studies of mice deficient in alternate *Lpaat* enzymes, which demonstrate a critical role for those enzymes in Kennedy Pathway steps in TAG biosynthesis [4,6]. However, body weights were similar between young *Lpaatδ*^−/−^ mice and their sex-matched wildtype (*Wt*) littermates [9]. Additionally, only epididymal white adipose tissue (WAT) differed in mass, although it was significantly larger in the *Lpaatδ*^−/−^ mice, due to impaired TAG lipolysis, rather than smaller, as would be expected if TAG synthesis was directly compromised by the loss of this enzyme [9]. Notably, while differences in adipose depot-specific lipid metabolism were identified in young *Lpaatδ*^−/−^ mice, this occurred without differences in whole-body metabolic measures, including energy expenditure, relative substrate oxidation rates, food intakes, or activity levels [9].

Our laboratory has an interest in the effects of aging on metabolic disease processes. To study how loss of *Lpaatδ*/*Agpat4* affects various conditions associated with aging, we generated a cohort of middle-aged *Lpaatδ*^−/−^ mice, and have recently reported on the behavioral phenotype of these animals [14]. In contrast to measures from younger mice [9], elevated anxiety-like behavior was evident with aging in this genotype [14], which suggested that other phenotypic parameters of *Lpaatδ*/*Agpat4* deficiency may also manifest with advancing age. We therefore questioned whether physiological and metabolic differences related to *Lpaatδ* gene deficiency may be present in 14-month-old *Lpaatδ*^−/−^ mice.

In the current work, we have investigated body weights and food intakes, ambulatory and locomotor activity, oxygen consumption (VO_2_) and carbon dioxide production (VCO_2_), respiratory exchange ratio (RER), and total energy expenditure (heat) in 14-month-old *Lpaatδ*^−/−^ mice and their *Wt* littermates. Results from this work indicate the development of a significantly altered metabolic phenotype in middle-aged male *Lpaatδ*^−/−^ mice that was not evident in previous studies on younger animals [9].

## 2. Materials and Methods

### 2.1. Animals

*Lpaatδ*^−/−^ and *Wt* littermate controls were generated and genotyped as previously described [8,9]. All experiments were performed in 14-month-old male mice, housed in a temperature and humidity-controlled environment, on a 12:12 h light/dark cycle. All animal procedures were approved by the University of Waterloo Animal Care Committee (AUPP#14-03, approved 29 April 2014; AUPP# 17-18, approved 27 June 2017; AUPP#18-05, approved 24 April 2018), and comply with guidelines of the Canadian Council on Animal Care.

### 2.2. Body Weight and Food Intake Measurements

Body mass and home-cage 24 h food intakes were measured in individually housed *Lpaatδ*^−/−^ and *Wt* mice with ad libitum access to standard rodent chow and water.

### 2.3. Locomotion and Indirect Calorimetry

Mice were single-housed in a Comprehensive Laboratory Animal Monitoring System (CLAMS; Accuscan Instruments Inc., Columbus, OH, USA) metabolic chamber for the determination of spontaneous locomotor behavior, oxygen consumption (VO_2_), carbon dioxide production (VCO_2_), respiratory exchange ratio (RER), and total energy expenditure (heat) over 24 h. Activity measures were recorded, including total locomotor activity, which is expressed as the number of infrared beam breaks along the x- (single beam) and y- (dual beam) axes (i.e., all beam breaks), and ambulatory activity, which is expressed as the number of infrared beam breaks along the y-axis only (i.e., consecutive beam breaks). Mice were housed in the cages for 26 h in total, wherein the first 2 h in the cages were excluded from all analyses to account for habituation to housing conditions.

### 2.4. Statistics

Results are expressed as means ± SEM. Student’s *t*-test (unpaired, two-tailed) was used to calculate statistical significance between the two groups. Statistical significance was accepted at *p* ≤ 0.05. Significance is reported as: * *p* < 0.05; ** *p* < 0.01; *** *p* < 0.001. Statistical analyses were performed using GraphPad Prism (GraphPad Software, San Diego, CA, USA).

## 3. Results

### 3.1. Body Weight and Food Intake

**Figure 1:**
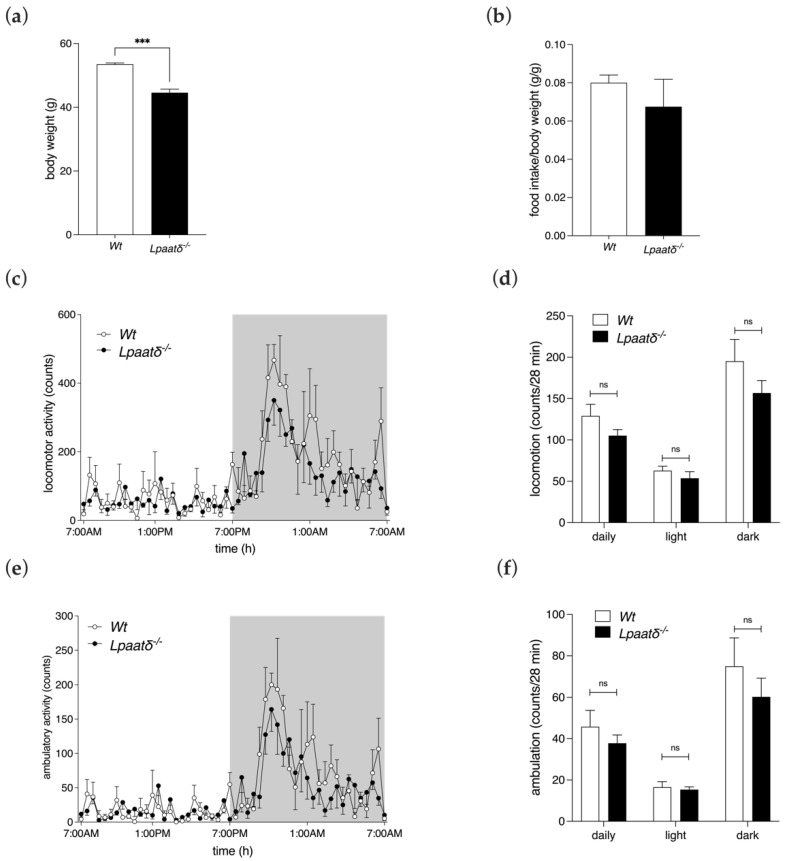
Middle-aged *Lpaatδ*^−/−^ mice have lower body weights but similar food intakes, and locomotor and ambulatory activity. Body weights (**a**) and daily food intakes (**b**) of 14-month-old male *Lpaatδ*^−/−^ mice and *Wt* littermates. Mice were individually housed in indirect calorimetry chambers for measurement of locomotor activity (**c**,**d**) and ambulatory activity (**e**,**f**) over a 24 h period, including 12 h light and 12 h dark photoperiods. Activity was expressed as the average sum of movement-induced infrared beam interruptions per 28 min interval, during the respective times. Shaded backgrounds in (**c**,**e**) denote the dark period, while unshaded backgrounds denote light periods. All data are expressed as means ± SEM (*n* = 4 per genotype). *** *p* < 0.001, ns = not significant for *Lpaatδ*^−/−^ vs. *Wt* mice.

We assessed body mass and food intake in single-housed 14-month-old male *Lpaatδ*^−/−^ and *Wt* littermate control mice. *Lpaatδ*^−/−^ mice had significantly lower average body weights than age-matched *Wt* mice (44.6 ± 2.16 g vs. 53.5 ± 0.84 g, *p* < 0.001, respectively) despite a lack of difference in mean daily food intake between the genotypes (Figure 1a,b). Since lower body weights could not be ascribed to lower energy intakes, this suggested that *Lpaatδ*^−/−^ mice may have greater energy expenditure.

### 3.2. Locomotion

Twenty-four-hour locomotor activity was measured by quantifying infrared (IR) beam breaks within the metabolic chamber and was analyzed to determine the average sum of IR beam breaks at each 28 min interval of recording, either throughout the entire period (24 h locomotor activity), or during the light or dark cycles. Mice are nocturnal, and therefore were more active during the dark cycle, as expected, and less active during the light cycle when their longer sleep cycles occur [15]. Average ambulatory and locomotor activity measures during the 12 h light cycle, 12 h dark cycle, and 24 h periods did not differ significantly between *Lpaatδ*^−/−^ and *Wt* mice (Figure 1c–f). Coupled with lower body weights, but also a lack of difference in food intakes, these findings suggest that differences in energy expenditure, respiratory gas exchange, and substrate utilization should be investigated for possible differences.

### 3.3. Metabolic Parameters

**Figure 2:**
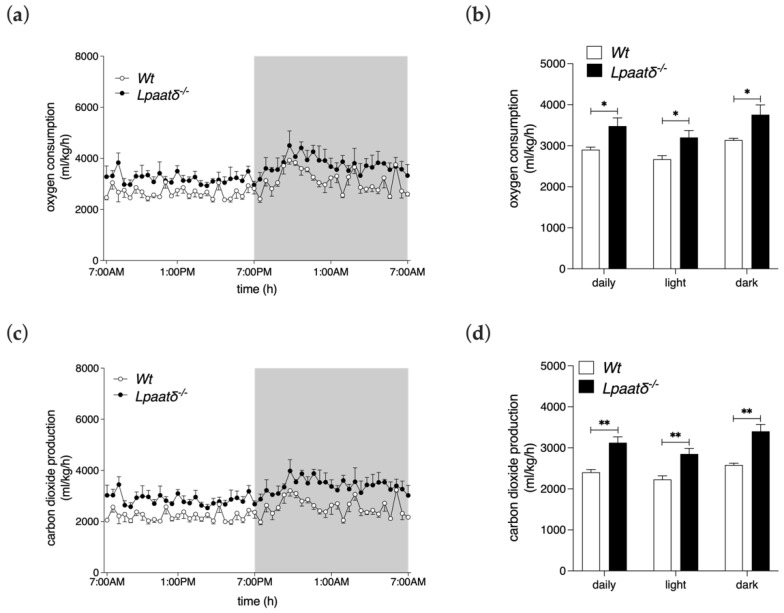
Fourteen-month-old *Lpaatδ*^−/−^ mice have elevated respiratory gas exchange rates. Mice were individually housed in indirect calorimetry chambers for 24 h. Shown are oxygen consumption rates (**a**,**b**) and carbon dioxide production rates (**c**,**d**) for *Lpaatδ*^−/−^ and *Wt* littermates, normalized to total body weights. Shaded backgrounds in (**a**,**c**) denote the dark period, while unshaded backgrounds denote light periods. All data are expressed as means ± SEM (*n* = 4 per genotype). * *p*  <  0.05, ** *p*  <  0.01 for *Lpaatδ*^−/−^ vs. *Wt* mice.

We recorded metabolic measures of mutant and control mice, and found evidence of significant differences between the genotypes in respiratory gas exchange. *Lpaat*δ^−/−^ mice consumed significantly more oxygen (VO_2_) (Figure 2a,b) and produced more carbon dioxide (VCO_2_) (Figure 2c,d) on average throughout the 24 h period examined. During either the light or dark photoperiod, VO_2_ consumption was ~20% higher in *Lpaat*δ^−/−^ mice, while VCO_2_ production was ~30% higher. 

**Figure 3:**
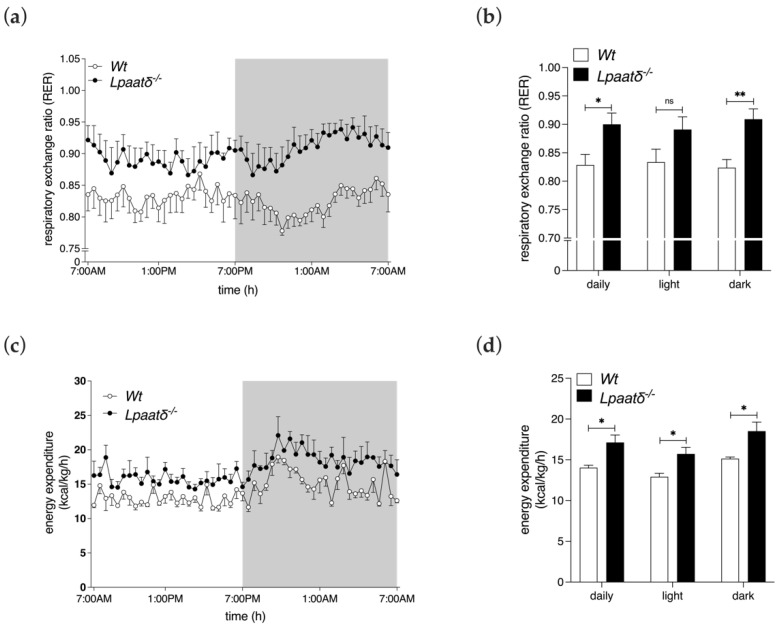
Respiratory exchange ratio and energy expenditure measures are higher in 14-month-old *Lpaatδ*^−/−^ mice than their *Wt* littermates. Mice were individually housed in indirect calorimetry chambers for 24 h. Measures of respiratory exchange ratio (RER) (**a**,**b**) and total energy expenditure (**c**,**d**) over 24 h were conducted. Energy expenditure measures were normalized to total body weights. Shaded backgrounds in line graphs denote the dark period, while unshaded backgrounds denote light periods. All data are expressed as mean ± SEM (*n* = 4). * *p*  <  0.05, ** *p*  <  0.01, ns = not significant for *Lpaatδ*^−/−^ vs. *Wt* mice.

Whole-body respiratory exchange ratio (RER) can be calculated from the ratio of VCO_2_/VO_2_ and used as an estimate of the relative contribution of carbohydrates and fats for energy production [16]. A slightly, but significantly higher average RER was recorded for *Lpaatδ*^−/−^ mice compared to their *Wt* littermates during the 24 h period (0.90 ± 0.04 vs. 0.83 ± 0.04 *Wt*, *p* < 0.05, respectively), and also specifically during the dark cycle (0.91 ± 0.04 vs. 0.82 ± 0.03, *p* < 0.01, respectively), indicating a greater contribution from carbohydrates relative to fats as the dominant substrate utilized by middle-aged *Lpaatδ*^−/−^ mice (Figure 3a,b).

The increase in average VO_2_ of 14-month-old *Lpaatδ*^−/−^ mice was expected to be associated with an increased metabolic cost. This was observed, since the average energy expenditure by *Lpaatδ*^−/−^ mice, determined as normalized heat production per h, was significantly higher during the 24 h period measured, and during the light and dark cycles, compared to measures recorded for *Wt* littermates (Figure 3c,d). Therefore, a greater basal metabolic rate, as a component of total energy expenditure, is likely to contribute, at least in part, to the lower body weight of 14-month-old *Lpaatδ*^−/−^ mice.

## 4. Discussion

LPAATδ catalyzes the formation of PA [10], a multifunctional lipid that is a central precursor for the biosynthesis of membrane and storage lipids [2], and is also a bioactive lipid that is active in cellular signaling [17]. Despite these important roles, global metabolic phenotyping of young (i.e., 9–12-week-old) *Lpaatδ*^−/−^ mice revealed no discernable abnormalities from their *Wt* littermates [9]. However, in our present work, which studied the same mouse model at 14 months-of-age, differences in metabolic parameters were evident. Middle-aged *Lpaatδ*^−/−^ mice had significantly lower body weights than their *Wt* littermates. Examination of principal regulators of energy balance revealed no significant differences in food intakes or locomotor activity between *Lpaatδ*^−/−^ and *Wt* mice, suggesting elevated basal metabolism may be a factor. Indeed, indirect calorimetry assessment found higher rates of oxygen consumption (VO_2_), carbon dioxide production (VCO_2_), and ultimately daily total energy expenditure (heat) in the middle-aged *Lpaatδ*^−/−^ mice. This increased metabolic cost was also accompanied by a greater reliance on carbohydrate oxidation for energy production.

In the current study, middle-aged *Lpaatδ*^−/−^ mice exhibited lower body weights, which contrasts with the phenotype of young *Lpaatδ*^−/−^ mice that had similar body weights, but a larger epididymal fat pad due to impaired lipolysis [9]. Adipose-specific metabolism was not examined in the *Lpaatδ*^−/−^ mice in the current study. However, we reported that *Lpaatδ*^−/−^ mice did have increased RER, which signifies a greater degree of carbohydrate oxidation relative to fat oxidation. An ongoing impairment of lipolysis in specific fat depots, as a consequence of LPAATδ deficiency, provides a plausible explanation for the higher RER in middle-aged mice, since restrained utilization of endogenous fat stores for metabolism may result in a greater reliance on carbohydrates to support energy production [18,19]. However, impaired lipolysis would be expected to contribute to increased adipose tissue fat mass and would thus be expected to result in higher body weights, which was not observed, suggesting either that higher lipolysis rates did not persist in middle-aged mice (and the greater RER measures resulted from a different lipid-glucose metabolic perturbation), or that increased energy utilization offset any adipogenic effect of lower lipolysis rates. 

Although not specifically measured in the current study, differences in RER suggest that perturbations in glycemic regulation may occur in older *Lpaatδ*^−/−^ mice. Defects in glucose use and regulation have been reported in both *Lpaatα*/*Agpat1* and *Lpaatβ*/*Agpat2* knockout mice. *Lpaatα*/*Agpat1* deficient mice are hypoglycemic, likely due in part to impaired gluconeogenesis [4]. Conversely, *Lpaatβ*/*Agpat2* knockout mice are hyperglycemic, reflecting the severe insulin resistance that is manifest in this model of lipodystrophy [6]. Since *Lpaatδ*^−/−^ mice exhibit a relatively mild phenotype relative to these other gene-deficiency models, it is likely that any related changes in glycemic control would be less severe. Regardless, our finding of significant differences in respiratory gas utilization and ratio suggests that further investigation of glycemic regulation in *Lpaatδ*^−/−^ mice is merited. 

*Lpaatδ*^−/−^ mice had higher VO_2_, VCO_2_, and total energy expenditure than *Wt* littermate controls, supporting the notion that an elevated basal metabolic rate may play a role in generating the lower body masses observed with age. Basal metabolic rate is largely determined by mitochondrial respiration rate, which in turn can be partially accounted for by the coupling efficiency of oxidative phosphorylation to ATP production [20]. Elevation of basal metabolism may be an indication of, and compensation for the relative dissociation of oxidative phosphorylation from ATP synthesis, since a greater amount of substrate needs to be oxidized, and thus oxygen consumed, to produce an adequate amount of ATP. Dysregulated PA metabolism may elicit pleiotropic effects on mitochondrial biology relating to membrane glycerophospholipid composition, biophysical properties, and various signaling processes [17,21,22], potentially leading to cellular and physiological consequences such as increased mitochondrial uncoupling and lower metabolic efficiency. 

Whether this occurs in middle-aged *Lpaatδ*^−/−^ mice remains to be investigated, but it is reasonable to hypothesize that LPAATδ-derived PA may be relevant to mitochondrial function, and its absence may be related to the elevated energy expenditure and resulting lower body weights seen in the middle-aged *Lpaatδ*^−/−^ mouse model. Although most phospholipid synthesis occurs in the ER, autonomous PA synthesis is known to occur in the mitochondria [21]. LPAATδ localizes to the outer mitochondrial membrane [3], and we have found that deficiency of *Lpaatδ* causes reductions in specific ethanolamine-containing phospholipids in brain mitochondria isolated from young mice, demonstrating a role for this enzyme in downstream mitochondrial phospholipid composition [8]. However, these changes were not associated with impairments in respiratory function and electron transport chain complex activities in mitochondria derived from young animals [8]. Since the regulation and maintenance of the mitochondrial lipidome is differentially modulated with age [23], it is likely that changes in the composition of mitochondrial membrane phospholipids due to disruption of the enzymatic activity of LPAATδ will be different in middle-aged compared to young *Lpaatδ*^−/−^ mice. Therefore, this should be investigated directly, with attention to related effects on mitochondrial functionality.

Beyond its precursor role, PA possesses inherent biological activity. The unique physicochemical properties of PA are conferred from the features that make it the simplest glycerophospholipid: a small anionic headgroup and two fatty acids esterified to the glycerol backbone, resulting in a negatively-charged molecule with cone-shaped geometry. Though PA is a quantitatively minor constituent of cellular and organellar membranes, these physicochemical properties promote protein–lipid interactions and induce negative (concave) membrane curvature, and thus can contribute to the control of various intracellular and cellular processes [24].

Evidence designates PA as a pro-fusion lipid. In vitro studies show that increased generation of PA on the mitochondrial surface results in mitochondrial aggregation and fusion [25,26], and conversely, conditions that decrease the mitochondrial content of PA induce mitochondrial fragmentation [27]. The mechanisms by which PA facilitates fusion are unclear, but may involve indirect regulation through the modulation of the activities of protein machinery which affect membrane dynamics further downstream, or by being further processed to other fusogenic lipids such as diacylglycerol [24]. Alternatively, PA may manipulate mitochondrial morphology directly by inducing local negative stress, promoting non-bilayer conformation required to restructure the membranes of merging or dividing mitochondria. The production of PA specifically by LPAATδ has been reported to mediate membrane restructuring during vesicle trafficking of the highly dynamic Golgi complex [12]. If LPAATδ has a designated method of operation, in the context of the hypothesis that different glycerolipid acyltransferase homologues produce different collections of PA species enabling the multivalent functionality of this lipid class, then LPAATδ may have a dual role in the Golgi complex and the mitochondria of regulating membrane dynamics. Studies on the dynamic channeling of lipid products from various LPAAT/AGPAT enzymes will be needed to fully understand the critical functions of these enzymes, and the non-redundant nature of their unique intracellular roles. 

This study joins a growing body of literature demonstrating interactions between genes involved in phospholipid metabolism and aging-dependent phenotypes. For example, mice deficient in phosphatidylethanolamine *N*-methyltransferase (PEMT), which is an important enzyme in the synthesis of phosphatidylcholine, have recently been studied at ages 9–10 weeks, and ~2 years [28]. By considering various tissues, at diverse timepoints, the critical importance of this enzyme in age-related metabolomic changes was delineated, highlighting the importance of considering various ages across the lifespan. Given that most disease processes have a temporal component, and that aging is the single greatest risk factor for the development of most chronic diseases, more research on older subjects, including middle- and older-aged knockout mice, is advised.

The reason for the appearance of metabolic differences in *Lpaatδ*^−/−^ mice at 14 months-of-age, but not at 2–3 months-of-age, was not apparent from this work. It is possible that these findings may indicate a cumulative effect of *Lpaatδ* deficiency, wherein a quantitatively small effect, or series of small effects that were not overt at a younger age, may accumulate over time to cause a large effect at a later age. There are many examples of cumulative age-related changes in both normal and pathophysiologic aging, and genetic variation can modulate outcomes [29,30,31]. Alternately, it is possible that *Lpaatδ* deficiency causes changes in cellular and organellar metabolism that are initially compensated [9], but later overcome homeostatic tendencies. Finally, it is possible that *Lpaatδ* deficiency causes acute changes in metabolic parameters that manifest at a particular age, in association with hormonal, or other age-related changes [32].

The major metabolic outcomes of *Lpaatδ* gene ablation in 14-month-old mice include a lower body weight, with higher daily energy expenditure and a greater reliance on carbohydrates for substrate metabolism. The production of PA is widely distributed among different enzyme family members. This results in diversity in the metabolic pathways and processes that are supported, and it is therefore likely that these differences contribute to the diversity in phenotypes associated with *Lpaat* gene ablation, including differences across the lifespan. Further molecular and cellular studies using *Lpaatδ*^−/−^ mice at older ages will help to clarify the in vivo function of this LPAAT family member. 

Aging is a normal process that is accompanied by significant physiological changes, and increased risk of pathophysiological changes. Metabolic differences in the current study were evident in older *Lpaatδ*^−/−^ mice, which were not detected in prior work on younger mice [9]. Future research in *Lpaatδ*^−/−^ mice should therefore investigate additional molecular, biochemical, and physiological processes, including aspects that were not overtly different in younger animals, such as mitochondrial respiration measures [7,8]. In particular, investigation of changes in systems that are highly susceptible to age-related alterations, such as the cardiovascular system, should be made. Given the significant resources needed to generate these models, this report highlights the importance of executing studies across the lifespan in order to fully elucidate the role of novel genes. 

## Data Availability

Data are available upon reasonable request to the authors.
